# Trastuzumab deruxtecan treatment for HER2-low metastatic scrotal extramammary Paget’s disease with bone marrow involvement: a case report

**DOI:** 10.3389/fonc.2026.1886979

**Published:** 2026-09-01

**Authors:** Yi Bao, Lei Zhang, Fenfen Jiang, Juanfen Mo

**Affiliations:** 1 The Department of Oncology, the Second Affiliated Hospital of Jiaxing University, Jiaxing, Zhejiang, China; 2 The Department of Medicine, the Second Affiliated Hospital of Jiaxing University, Jiaxing, Zhejiang, China; 3 Department of Geriatric Medicine, the Second Affiliated Hospital of Jiaxing University, Jiaxing, Zhejiang, China

**Keywords:** bone marrow involvement, extramammary Paget’s disease, HER2, metastatic, trastuzumab deruxtecan (T-DXd)

## Abstract

Scrotal Extramammary Paget’s disease (EMPD) is a rare cutaneous adenocarcinoma commonly confined to the intraepidermal region, with potential for dermal invasion and metastasis. This report describes a rare case of metastatic scrotal EMPD with bone-marrow involvement. The patient presented with localized pruritic scrotal lesions accompanied by systemic symptoms resulting from distant metastases to the lymph nodes and multiple bones, as well as compromised hematopoiesis caused by tumor cell infiltration of bone marrow. The diagnosis was confirmed by histopathological examination of biopsy specimens from the scrotal lesion, local lymph nodes, and bone marrow. Immunohistochemistry (IHC) revealed HER2 status as 2+ with a negative fluorescence *in situ* hybridization result. Although the patient presented with a heavy tumor burden involving the bone marrow, he appeared to derive benefit from trastuzumab deruxtecan (T-DXd), a HER2-directed antibody-drug conjugate, and observed disease stability during the treatment period. This case suggests the potentially aggressive biological behavior of EMPD and demonstrates that targeted therapeutics, such as T-DXd, may achieve disease control in cases with low HER2 expression. These findings emphasize the need for further research to establish optimal treatment strategies for metastatic EMPD.

## Introduction

Extramammary Paget’s disease (EMPD), particularly in the scrotum, represents a rare cutaneous malignancy characterized by the common presence of adenocarcinoma within the epidermis (1). EMPD can also invade the dermis and metastasize to regional lymph nodes (LNs) and distant organs, a condition classified as invasive and metastatic EMPD (2). This case reports details an exceptional instance of scrotal Paget’s disease complicated by bone marrow metastasis, a rare occurrence in the literature. The patient presented with localized pruritic scrotal lesions accompanied by systemic symptoms resulting from distant metastases to the lymph nodes and multiple bones, as well as compromised hematopoiesis caused by tumor cell infiltration of bone marrow. The diagnosis of stage IV scrotal Paget’s disease was confirmed by biopsies of the right scrotal region and bone marrow. The patient’s observation of radiological disease control with Trastuzumab deruxtecan (T-DXd), an antibody-drug conjugate targeting HER2, illustrates the significance of precision medicine in the management of such atypical malignancies, particularly in cases where HER2 expression is low (3). This case contributes to the broader understanding of scrotal Paget’s disease, particularly regarding the potentially aggressive biological behavior of EMPD. These results suggest that targeted therapeutics, such as T-DXd, may provide disease control in cases with low HER2 expression; furthermore, the case demonstrates the need for further research to establish optimal treatment strategies for metastatic EMPD.

## Case presentation

A 68-year-old non-smoking Chinese male, who had a history of Paget’s disease in the right scrotal region eight years ago, experienced mild back pain in early November 2024. He presented to a local hospital where a series of tests were conducted based on his clinical presentation. The patient initially received medical care at a different local hospital. He was unable to provide his comprehensive medical records detailing his initial diagnosis and treatment, but he recalled only that he achieved a remission after photodynamic therapy. We also obtained his textual hematoxylin-eosin staining reports recording EMPD *in situ* of the right scrotum in March 2018.

### Diagnostic assessment

**Figure 1:**
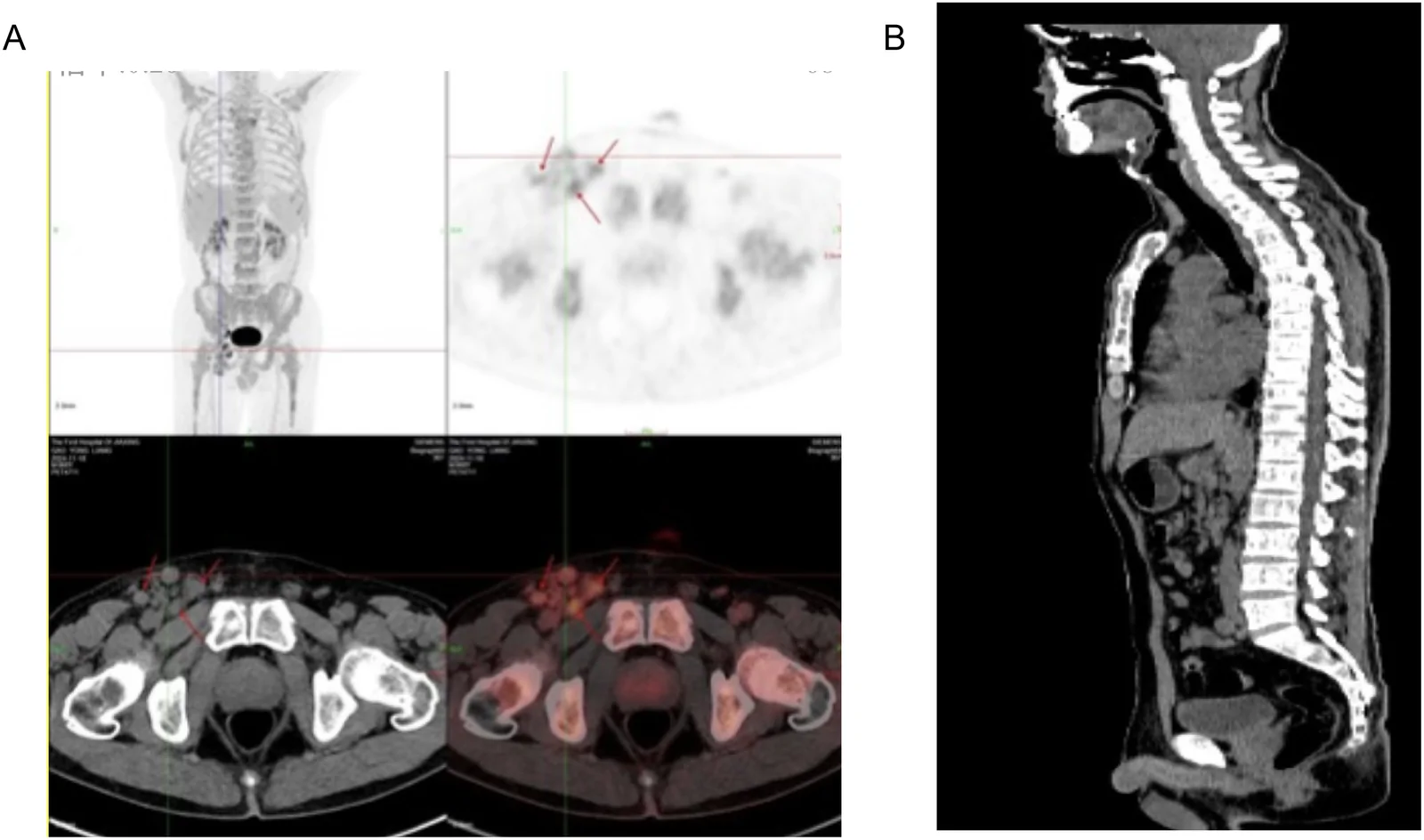
PET-CT showed localized thickening of the right scrotal skin with mildly increased patchy FDG metabolism, suggestive of a malignant skin lesion; multiple enlarged lymph nodes with increased FDG metabolism in the right inguinal region, right iliac vessel area, bilateral pelvic walls, bilateral common iliac arteries, para-aortic region, and left supraclavicular area, and multiple osteolytic lesions within the visualized field **(A, B)**.

**Figure 2:**
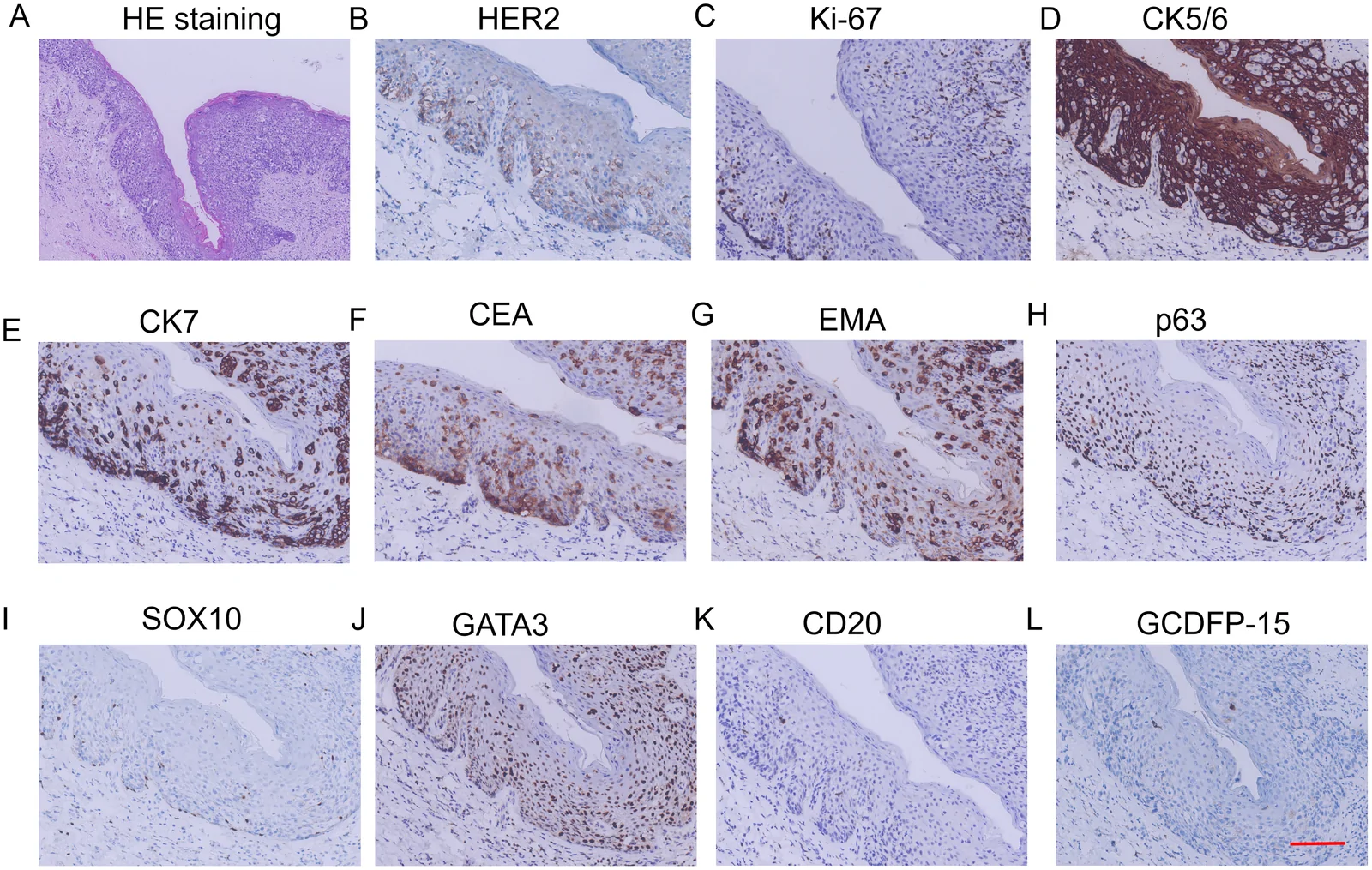
HE and IHC staining images: **(A)** HE staining; **(B)** HER2 (2+); **(C)** Ki-67 (30%+); **(D)** CK5/6 (-); **(E)** CK7 (+); **(F)** CEA (+); **(G)** EMA (+); **(H)** p63 (-); **(I)** SOX10 (-); **(J)** GATA3 (+); **(K)** CD20 (-); **(L)** GCDFP-15 (-). × 200, scale bar: 100 *µ*m.

Laboratory tests revealed elevated tumor biomarkers, with Carbohydrate Antigen 72-4 (CA72-4) levels exceeding 500 IU/ml and Carcinoembryonic Antigen (CEA) at 24.1 ng/ml. An abdominal CT scan revealed multiple osteolytic bone lesions, as well as enlarged lymph nodes in the retroperitoneum, around the iliac vessels, and in the right inguinal region (**Figure 1a**). Further Positron Emission Tomography-Computed Comography (PET-CT) scans revealed localized skin thickening on the right scrotal region with increased Fludeoxyglucose[18F] (18F-FDG) uptake, multiple enlarged lymph nodes around the right groin iliac vessels, bilateral common iliac arteries, abdominal aorta, and left supraclavicular area, together with extensive osteolytic bone destruction and diffuse increased 18F-FDG uptake in the bone marrow cavity (**Figures 1A, B**). A bone marrow biopsy confirmed the infiltration of metastatic tumor cells. The patient presented to our institution for further management on Nov 20, 2024. Biopsy of the right scrotal region revealed Paget’s disease characterized by dermal infiltration of tumor cells, while biopsy of the right inguinal lymph nodes confirmed the presence of metastatic tumor cells. Immunohistochemistry results demonstrated expression of HER2 (2+) in the tumor cells (**Figure 2**); however, a fluorescence *in situ* hybridization (FISH) test revealed a negative result for HER2 gene copy number amplification. Regrettably, the patient declined a repeat bone marrow biopsy at our institution; consequently, immunohistochemical analysis of the bone marrow specimen could not be performed. Additionally, the absence of cystoscopy for urinary tract evaluation and gastrointestinal assessment, including colonoscopy, represents a diagnostic limitation. However, based on the available evidence, the clinical diagnosis for the patient was stage IV Extramammary Paget’s disease.

### Therapeutic intervention

According to ECOG performance status, a score of 2 was initially assessed. At the patient’s request, he was discharged and transferred to a top-ranked hospital specializing in cancer treatment in Zhejiang province of China subsequently. Unfortunately, the patient was admitted to our hospital on March 13, 2025, due to severe thrombocytopenia (platelet count: 16 x10^9/L). According to the medical records provided by the patient, he was given three cycles of 200 mg of Pucotenlimab (an anti-PD-1 immune checkpoint inhibitor) and 60 mg of Disitamab vedotin (a HER2-directed antibody-drug conjugate drug) since December 26, 2024. Initially, it was difficult to determine whether the severe thrombocytopenia was caused by the immunotherapy-related adverse events or the progression of Paget’s disease due to tumor cell infiltration in the bone marrow. Platelet transfusions are administered to the patient immediately in preventing life-threatening bleeding. However, the patient exhibited signs of allergic shock during the platelet transfusion, which led to the immediate termination of the transfusion. His platelet counts was still maintained at sever low levels (21 x10^9/L). Due to concerns about the risk of bleeding, the patient refused the bone marrow biopsy. After a multidisciplinary team (MDT) discussions together with experienced haematologists and blood transfusion specialists, glucocorticoids at 1.5 mg/kg were administered intravenously daily to alleviate possible immunotherapy-induced thrombocytopenia. Meanwhile, other causes of severe thrombocytopenia were investigated. Fortunately, the platelet count of the patient was gradually increased, and reached 67 x 10^9/L after one week of administration of glucocorticoids and dosage reduction during follow-up. Therefore, we have considered that the severe thrombocytopenia was likely caused by the side effects of immunotherapy. However, given the patient’s complex history of systemic treatment, exposure to HER2-directed ADC, and bone-marrow involvement of cancer cell metastasis, determining the exact etiology was complicated. Since the patient decided to continue his anti-cancer treatment at our hospital, only 60 mg of Disitamab vedotin was administered on 3 April 2025. The patient again exhibited signs of anaphylactic shock during the infusion of Disitamab vedotin. The infusion was stopped, and epinephrine was administered immediately. He recovered and discharged from our hospital three days later. The patient returned seeking follow-up treatment three weeks later. According to Eastern Cooperative Oncology Group (ECOG) performance status (PS), a score of 3 was assessed and after fully discussion with the patient, Trastuzumab deruxtecan (T-DXd) was administered at a reduction dose of 200 mg (4.8 mg/kg) on April 24, 2025 and every 3 weeks a cycle in follow up. A reduced dose of 4.8 mg/kg of T-DXd because the patient has an ECOG score of 3 before T-DXd assessment. Additionally, T-DXd is available in 100 mg/vial. If calculated according to the normal dosage (5.4 mg/kg), there would be significant waste, imposing a considerable financial burden on the patient. Fully informed consent regarding off-label drug use was obtained. Due to a prior anaphylactic reaction to disitamab vedotin, we were also concerned about hypersensitivity reactions to T-DXd, and therefore, before T-DXd infusion, dexamethasone and promethazine hydrochloride were routinely given as premedication. T-DXd, an antibody-drug conjugate, consists of an anti-HER2 antibody, a cleavable linker, and a highly potent topoisomerase I inhibitor (4). This drug, once released inside tumor cells, can penetrate neighboring HER2-low expressing or even negative cancer cells, which is critical for heterogeneous tumors with low levels of HER2 expression (4).

### Follow-up and outcomes

**Figure 3:**
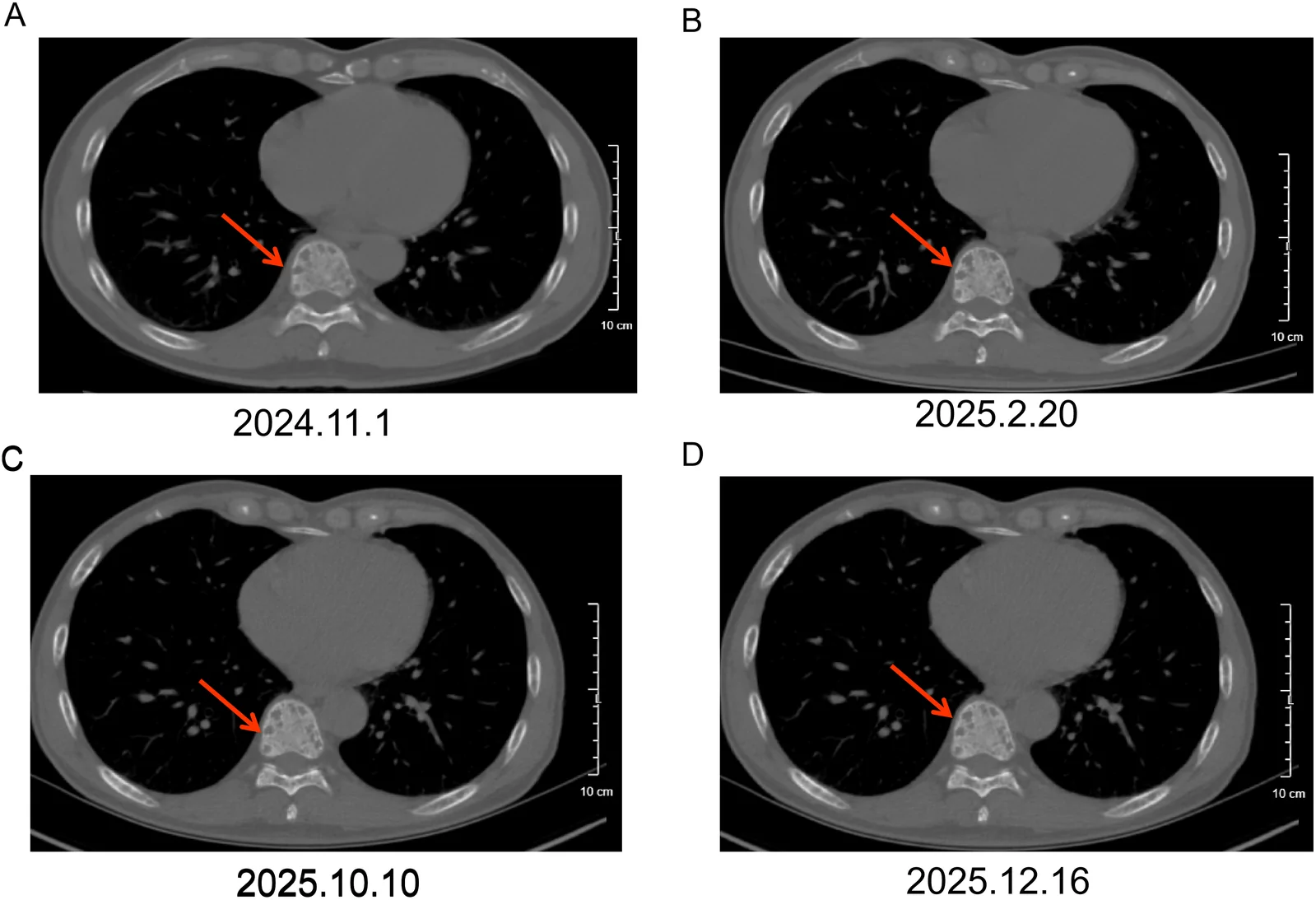
A series of chest CT scans were recorded osteolytic lesions of the thoracic vertebrae that maintained stable conditions **(A-D)**.

**Figure 4:**
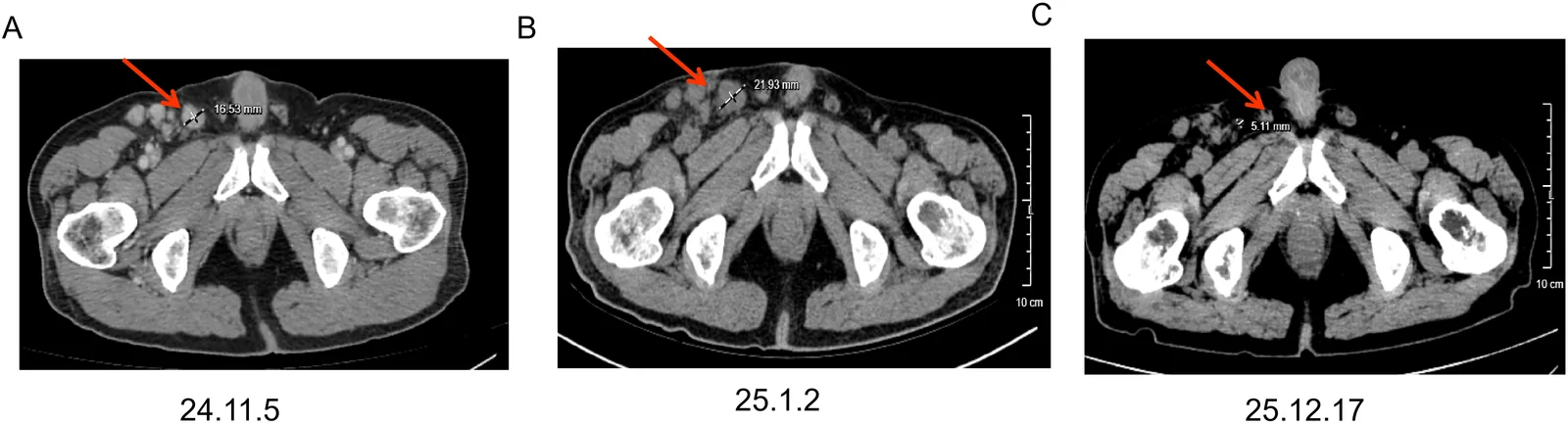
The abdominal CT scan showed multiple enlarged lymph nodes in the right inguinal, bilateral iliac vessel, right pelvic wall, and retroperitoneal regions. Compared to the abdominal CT scans presented in **(A, B)**, which were recorded on November 4, 2024 and January 2025, the transverse diameter of one representative lymph node in the right inguinal region decreased from 16.53 mm and 21.93 mm, respectively, to 5.11 mm on December 17, 2025 **(C)**.

Notably, during the T-DXd treatment period, the patient did not experience an anaphylactic reaction. His ECOG performance status (PS) was stable at 2 following routine pre-treatment assessments in each cycle. Drug-related side effects were routinely monitored by assessing symptoms (such as nausea, vomiting, and fatigue), hematologic parameters, hepatic and renal function tests etc. Additionally, pulmonary symptoms and imaging (chest CT scans for interstitial lung disease or pneumonitis; Color Doppler Echocardiography for cardiac function) were utilized to facilitate early detection of Treatment Emergent Adverse Events (TEAEs), including neutropenia, anemia, and a decline in left ventricular ejection fraction (LVEF). Platelet counts were maintained above 100 x 10^9/L during subsequent treatment for most of the time during the T-DXd safety assessment window. On August 11, 2025, the patient presented with thrombocytopenia, with a platelet count of 36 x 10^9/L. We were unable to determine whether this was an adverse effect of T-DXd or a COVID-19 infection, as confirmed by laboratory tests. The patient presented with rhinorrhea, cough, and mild fever at that time, alongside Grade 1 interstitial lung disease. We could not definitively exclude T-DXd-induced thrombocytopenia, nor could we rule out an association between SARS-CoV-2 infection and thrombocytopenia. He gradually recovered following treatment with simnotrelvir/ritonavir combined with glucocorticoids at 1.0 mg/kg, and therefore we attribute these abnormalities likely to sequelae of the COVID-19 infection. T-DXd was continued in the follow-up treatment. Routine laboratory tests assessing hematologic toxicity and hepatic function are provided in **Supplementary Table 1**. Adverse events graded according to the Common Terminology Criteria for Adverse Events (CTCAE) are listed in **Supplementary Table 2**. However, comprehensive images were not acquired immediately prior to the first T-DXd cycle, and subsequent follow-up imaging was conducted at irregular intervals. Here, a series of chest CT scans were provided, including osteolytic lesions of the thoracic vertebrae that maintained stable conditions (**Figure 3**). On December 17, 2025, the abdominal CT scan revealed that a reduction in the size of multiple enlarged lymph nodes in the right inguinal, bilateral iliac vessel, right pelvic wall, and retroperitoneal regions, compared to the abdominal CT scan presented in **Figures 4A, B** which were recorded on November 4, 2024 and January 2025 respectively (**Figure 4C**) illustrated decreased lymph nodes in the right inguinal region). These findings present a definitive assessment of radiological disease control, as imaging was not performed immediately before the first T-DXd administration and subsequent evaluations occurred at irregular intervals; therefore, a formal Response Evaluation Criteria In Solid Tumors (RECIST) assessment of treatment response cannot be reliably performed. The last treatment was given on January 31, 2026, and a total of 12 cycles of T-DXd were administered at our medical center. However, his platelet counts declined precipitously, reaching 27 × 10^9/L on February 25, 2026. Consequently, T-DXd therapy was discontinued completely at our medical center. We also present a comprehensive diagnosis and treatment timeline in **Supplementary Figure 1**.

## Discussion

Paget’s disease of the scrotum is a rare malignant tumor that can be treated by surgical intervention or non-surgical treatment such as photodynamic therapy, if surgical therapy is not appropriate (5, 6). However, the absence of effective targeted therapy significantly impairs the quality of life in these rare patients experiencing local recurrence or distant metastasis (2).

Regional lymph node involvement is frequently the earliest sign of metastasis of EMPD and is a key negative prognostic factor (7). In the present case, the patient was diagnosed with recurrent scrotal EMPD accompanied by multiple metastases to regional lymph nodes, bone, and bone marrow, which were confirmed through comprehensive radiological imaging (such as PET-CT) and biopsies. The rarity of this disease complicates its diagnosis and impedes the effective management strategies (2). Patients with metastatic EMPD are often candidates for conventional chemotherapy, such as docetaxel, which is commonly administered (8). Other treatment methods, including targeted therapy and immune checkpoint inhibitors, have been investigated to improve overall survival (9, 10).

Traditionally, human epidermal growth factor receptor 2 (HER2)-targeted therapies for tumors such as gastric and breast cancers have been based on an immunohistochemistry (IHC) score of 3+ or positive fluorescence *in situ* hybridization FISH/*in situ* hybridization (ISH) results (11). The clinical definition of HER2-low status relies on the standard IHC/ISH approach, characterized by an HER2 IHC score of 1+ or 2+ with a negative ISH assay (12). Notably, in a phase II single-arm clinical trial, promising data were reported in a small cohort of Japanese patients with HER2-positive metastatic EMPD who received combination therapy with trastuzumab and docetaxel (13). A systematic review of 7 cases reported an overall response rate (complete and partial responses) of 85.7% following trastuzumab-based therapy in HER2-positive metastatic EMPD (7). These findings suggest that HER2-guided personalized treatment may be a promising therapeutic approach for metastatic EMPD. However, there remains a lack of randomized, open-label, multicenter, phase III prospective studies HER2-positive cases on the prognosis of patients with metastatic EMPD. In the present case, the IHC score of HER2 was 2+, while the FISH test was negative, categorizing the patient as having “low HER2 expression”. He initially received immunotherapy in combination with a HER2-directed ADC. However, the patient developed severe thrombocytopenia during treatment. Subsequently, the clinical course was further complicated by anaphylactic shock during the platelet transfusion. The patient’s severe thrombocytopenia was alleviated by corticosteroids, suggesting it may be primarily attributed to immunotherapy. However, determining the exact etiology of severe thrombocytopenia was complicated given the patient’s extensive history of systemic treatment, exposure to a HER2-directed ADC, and bone marrow involvement. Consequently, severe thrombocytopenia may be attributed to multiple factors. During subsequent treatment with T-DXd, another HER2-directed ADC, the patient exhibited radiological disease control for more than eight months, suggesting that HER2 status may play a critical role in metastatic EMPD. The safety profile was regularly monitored during T-DXd treatment using the CTCAE grading system, with specific attention to adverse events such as drug-related interstitial lung disease, pneumonitis, and neutropenia (**Supplementary Table 2**). Unfortunately, the patient completely terminated his treatment with T-DXd at our hospital on February 25, 2026 due to severe thrombocytopenia again. We were unable to determine whether the severe thrombocytopenia was attributable to a drug-related adverse event or disease progression secondary to bone marrow metastasis. The attribution of thromocytopenia and interstitial lung disease should remain cautious because several potential contributing factors were present, including bone marrow infiltration, previous systemic treatments, COVID-19 exposure, and T-DXd exposure. Additionally, severe thrombocytopenia following the last cycle of T-DXd administration still warrants caution regarding associated safety outcomes.

Antibody-drug conjugates (ADCs) are mAbs covalently bound to potent cytotoxic agents. T-DXd, which is approved for HER2-expressing breast and gastric cancers and HER2-mutant non-small-cell lung cancer. T-DXd is an ADC composed of trastuzumab, a cleavable linker, and the topoisomerase I inhibitor deruxtecan. Topoisomerase I inhibitors such as irinotecan (CPT-11) bind to topoisomerase I-DNA cleavable complexes and stabilize them, inducing double-strand DNA breaks and apoptosis (14). Clinical trials, such as DESTINY-Breast04, showed that T-DXd improved overall survival (OS) compared with traditional chemotherapy for patients with HER2-low (IHC 1+ or IHC 2+/*in situ* hybridization-negative) metastatic breast cancer and metastatic gastric cancer (15, 16). Results from exploratory cohorts of DESTINY-Gastric01 suggested a potential benefit from T-DXd in patients with HER2-low (IHC 2+/*in situ* hybridization-negative) gastric cancer (16, 17). In an open-label phase II study, the DESTINY-PanTumor02 Phase II Trial evaluated T-DXd (5.4 mg/kg once every 3 weeks) in patients with HER2-expressing (IHC 3+/2+) locally advanced or metastatic solid tumors who had received ≥1 prior systemic treatment or had no alternative treatments. The median PFS was 6.9 months (95% CI, 5.6 to 8.0), and the median OS was 13.4 months (95% CI, 11.9 to 15.5) (18);these findings support the potential role of T-DXd as a tumor-agnostic therapy for patients with HER2-expressing solid tumors, especially in the HER2 IHC 3+ population (18).

This case demonstrates the extension of this therapeutic approach to exceedingly rare scrotal Paget’s disease, exemplifying HER-directed ADC therapy across different tumor types. The drug appears to have stabilized the primary and metastatic lesions and achieved possible disease control, accompanied by improved hematopoietic function, which may be explained by the observed recovery of platelet counts. The management of this case provides the possible transition from traditional chemotherapy to a novel ADC targeting HER2-low expressing EMPD. It is important to note that evidence regarding HER2-low EMPD remains extremely limited.

### Limitation of the case

First, data continuity was compromised because the patient received diagnosis and treatment at different hospitals. Second, a bone marrow biopsy was performed at an external hospital; however, the patient rejected bone marrow biopsies at our medical institution. Therefore, immunohistochemical analysis of bone marrow biopsy specimens was not performed at our institution. Third, the patient had markedly high CA72–4 and elevated CEA levels accompanied by extensive nodal and bone osteolytic destruction and bone marrow infiltration; therefore, cystoscopy with urologic evaluation, colonoscopy with gastrointestinal evaluation, and prostate assessment are valuable to exclude secondary malignancy. Finally, imaging data were not acquired at regular intervals. The absence of a follow-up PET-CT evaluation represents a significant limitation, particularly given the patient’s extensive bone metastases.

In summary, this case contributes to the broader understanding of scrotal Paget’s disease and demonstrates the need for further research to establish optimal treatment strategies for metastatic EMPD.

## Data Availability

The raw data supporting the conclusions of this article will be made available by the authors, without undue reservation.
